# Invited commentary: The need for human genetics and genomics in dental school curricula

**DOI:** 10.1002/mgg3.216

**Published:** 2016-03-17

**Authors:** P. Suzanne Hart, Thomas C. Hart

**Affiliations:** ^1^ National Human Genome Research InstituteNational Institutes of HealthBethesdaMaryland; ^2^ American Dental Association FoundationVolpe Research CenterGaithersburgMaryland

There are currently 67 dental schools in the United States and Puerto Rico. According to the 2014 edition of the *Official Guide to Dental Schools For Students Entering in Fall* 2015 published by the American Dental Education Association (www.adea.org), no dental schools currently require genetics prior to admission. Only one school, the University of Florida, requires one semester of molecular biology or genetics for admission. When last surveyed in 2001, only eight of 53 dental schools had a formal course in human genetics in their curricula (Dudlicek et al. 2004). Only one school did not respond to the survey. Despite calls from a variety of individuals and professional organizations that genetics should be an integral part of dental school curricula (Wright and Hart 2002; Behnke and Hassell 2004; Collins and Tabak 2004; Johnson et al. 2008; Slavkin 2014), little progress has been made to improve the teaching of human genetics to dental students. With this in mind, we once again call for dental schools to include human genetics as a formal course in their curricula.

A search of the scientific literature reveals how the contribution of genetic factors to missing or misshapen teeth, cleft lip/palate, oral cancer, caries, periodontal disease and other oral pathologies and conditions continues to expand. The effects that systemic disorders can have on an individual's oral health are also well known. Gingival hyperplasia can be an isolated condition, part of a syndrome, or a side effect of certain medications. If a side effect of medication, it is reversible simply by stopping the drug. Inherited forms require surgical resection. Thus, a dentist needs to able to take a family and medical history to distinguish the forms. Associations have been made between enamel defects and kidney disease (Jaureguiberry et al. 2013), between missing teeth and colon cancer (Lammi et al. 2004), and between microdontia and deafness (Riazuddin et al. 2011). Knowing whether a dental phenotype is an isolated finding or is associated with other systemic manifestations with broader healthcare implications can lead to appropriate referrals. Dentists clearly have patients with genetic disorders in their practice. These genetic disorders may or may not have an impact on their oral health. These include Mendelian traits such as amelogenesis imperfecta and cystic fibrosis, cytogenetic disorders such as Smith–Magenis and Williams syndromes, as well as multifactorial traits such as cleft lip/cleft palate or diabetes. The last decade has seen an explosion in the number of genes associated with craniofacial development and diseases. Currently, mutations in at least seven genes are associated with amelogenesis imperfecta, a disorder of qualitative or quantitative defects of enamel (Table 1).

**Table 1:** Genes that Cause Amelogenesis Imperfecta

Gene	Chromosome	Mode of inheritance
Kallikrein 4 (*KLK4*)	19	Autosomal Recessive
Enamelin (*ENAM*)	4	Autosomal Dominant and Autosomal Recessive
*WD Repeat Domain 72 WDR72*	15	Autosomal Recessive
Matrix Metalloproteinase 20 (*MMP20*)	11	Autosomal Recessive
*FAM20A*	17	Autosomal Recessive
*FAM83H*	8	Autosomal Dominant
Amelogenin (*AMELX*)	X	X‐linked

There are several reasons why genetics and genomics should be included in dental school criteria beyond the obvious value of making a dental diagnosis. Being able to take a family history to construct a three‐generation pedigree is crucial. While one should not discount the psychological or financial burden of missing or malformed teeth, genetic disorders may also have extraoral health consequences. For example, dentinogenesis imperfecta (DI) may occur as an isolated finding or as part of a syndrome such as osteogenesis imperfecta (OI), which is associated with bone fragility and hearing loss (Hart and Hart 2007). Mild type 1 OI may be mistaken for isolated DI (Pallos et al. 2001). Making a correct diagnosis is crucial for a discussion of phenotypic consequences, management, and genetic counseling for recurrence risks. Enamel renal syndrome is an autosomal‐recessive disorder due to mutations in the *FAM20A* gene. The combined dental features of amelogenesis imperfecta and gingival hyperplasia are highly suggestive of this disorder. The renal phenotype, nephrocalcinosis, is typically clinically asymptomatic in children. Dentists should refer individuals with the oral phenotype to nephrologists for evaluation (Jaureguiberry et al. 2013). Mutations in the *ANIX2* gene have been associated with oligodontia and colorectal cancer (Lammi et al. 2004). Although not all patients with oligodontia have an increased risk of colorectal cancer, a history of colorectal cancer in the blood relatives of an oligodontia patient should prompt consideration of referral to either a genetic counselor or clinical geneticist for further evaluation.

Dentists also need to have an understanding of genetics to appreciate the issues surrounding genetic testing. More than 3000 genetic tests currently available encompass a variety of genetic disease types. The majority of these tests for Mendelian and cytogenetic conditions are clinically valid and useful; however, they must be used properly. Unfortunately, some of the tests offered, particularly tests for small effect SNP variants marketed to predict risk for common, complex diseases are neither clinically valid nor clinically useful (SACGT 2000; Diehl et al. 2015; Ioannidis 2015). The dental provider will need to be able to evaluate these tests and decide whether or not to incorporate them into their clinical practice. In many cases, this will involve consultation with non‐dental health care providers, including geneticists and genetic counselors.

As our understanding of genetics and the role of genetic factors in normal and abnormal development increases, it is imperative that dental students are taught more than just Mendelian and cytogenetic disorders. Dentists need to understand multifactorial conditions as well as appreciate how environmental exposure to microbial, viral, pharmacologic, diet, smoking, and other factors can affect the genetic and epigenetic landscape (Ambatipudi et al. 2016). This is particularly true for the dental care providers where smoking and the oral microbiome have a direct impact on the development of caries, periodontal diseases, and other oral pathologies.

The patient‐educator model, in which patients tell their own stories to dental students, is a way to better engage learning and retention of knowledge. Although used extensively in the training of medical students and residents, this is a fairly new practice in dentistry (Renard et al. 2015). The authors of this study report that patient‐educators reinforce the importance of understanding basic science, including genetics, in the students’ future dental practice. Three years after experiencing patient‐educator teaching, 83% of dental students correctly diagnosed the genetic condition in a case‐based scenario. Another important facet of patient‐educators is bringing to light the psychosocial aspects of a particular disorder. Given the huge cosmetic side of dentistry, this psychosocial aspect should not be overlooked. Studies have shown that students often learn faster and are more empathetic when patient‐educators are involved (Renard et al. 2015). We believe that incorporation of patient‐educators should be adopted at some level in all dental schools.

Health care providers cannot work in a vacuum. The dental practitioner needs to know where to go to look for genetic resources. When appropriate, the dentist should be part of the personalized medicine team. They need to be able to speak the language and understand basic concepts in order to interact with other members of the team in a meaningful way. This lack of genetics education limits the dentist's ability to interact with the larger health care team, further isolating dentistry from other health care disciplines. This isolation is confounded by separate health care records and distinct reimbursement systems (Regier and Hart 2016).

In summary, dental graduates should have the basic genetics knowledge and skills to provide the educational foundations for understanding and applying genetics to clinical practice. While the genetic core competencies for such knowledge, skills, and attitudes will need to be developed for the dental profession, examples have been developed by others, including recommended core competencies in genetics for all health care professionals (Jenkins et al. 2001; NCHPEG 2007). The failure of dental schools to incorporate human genetics into their curricula is a failure to their students who will surely encounter patients with genetic disorders in their practice. Students without adequate training in genetics will not be prepared to effectively diagnose patients, adequately evaluate new therapies or tests based upon genomic information, nor work collaboratively with other members of the health care team. In the end, it will be the patients who suffer.
